# Self‐Care Practice in the Management of Hypertension Among Adults in Africa: Findings From a Scoping Review

**DOI:** 10.1002/hsr2.72457

**Published:** 2026-04-29

**Authors:** Abiola Adeosun, Hafiz T. A. Khan, Muili Lawal

**Affiliations:** ^1^ College of Nursing, Midwifery and Healthcare University of West London UK; ^2^ Faculty of Basic Medical Sciences Lead City University Ibadan Nigeria; ^3^ Oxford Institute of Population Ageing University of Oxford Oxford UK

**Keywords:** adults, Africa, hypertension, review, self‐care

## Abstract

**Background and Aims:**

Effective self‐care for hypertension spans medication adherence and lifestyle practices (dietary salt reduction, physical activity, weight control, smoking/alcohol moderation). This scoping review summarizes self‐care strategies employed by adults with hypertension in Africa and identifies factors influencing their self‐care practices.

**Methods:**

Using the PRISMA‐ScR scoping review framework, following the Joanna Briggs Institute manual for scoping review, out of 8084 studies collected from CINAHL, PUBMED, ScienceDirect, PsycINFO, EMBASE, and Google Scholar, 18 studies consisting of 15 quantitative and 3 qualitative were included using the Population, Concept, and Context framework.

**Results:**

Of 8084 records (43 duplicates removed), 8041 titles/abstracts were screened; 134 reports were sought, 118 full texts assessed, and 18 studies included (15 quantitative; 3 qualitative) from Ethiopia (8), Nigeria (3), Ghana (2), and 1 each from Tanzania, Uganda, Egypt, Malawi, and Tunisia. Most studies reported low to moderate adherence across lifestyle domains; medication adherence varied, but inadequate dietary salt control, physical inactivity, and limited weight management were frequent. Education/knowledge, urban residence, and social support were commonly associated with better self‐care, whereas medicine stock‐outs, self‐prescription, traditional remedies, stigma around weight loss, and khat chewing emerged as barriers. Measurement approaches varied, with H‐SCALE most frequently used.

**Conclusion:**

Among adults attending African health facilities, self‐care practices are suboptimal, particularly for lifestyle behaviors, especially dietary management, weight control, and aerobic exercise. Interventions that bolster patient education, reliable medicine supply, social support, and culturally‐sensitive counseling may improve adherence. Future research should standardize measurement and better report context to guide implementation.

## 1 Introduction

Self‐care refers to an individual's actions to prevent or limit illness and promote health [1]. The World Health Organization (WHO) recommended self‐care to improve outcomes in the management of ailments, especially chronic diseases such as hypertension. In developed countries, patients are mostly encouraged and provided awareness about self‐monitoring, self‐behavior (i.e., lifestyle management), self‐titration of medication, and adherence to regimens [2]. The government of the United Kingdom and other developed countries promote self‐reliance, as over 50% of healthcare services are provided through supported self‐management and medications [3, 4]. A United States study on assisted self‐monitoring via online and home care of hypertension reported a significant reduction in blood pressure over 12 months [3].

Compliance with self‐care practice among hypertensive patients helps to control blood pressure, prevent complications, and reduce mortalities associated with the disease [5]. Effective self‐care practices require altitude, knowledge, discipline, and commitment to manage the disease and achieve healthy living [6]. Studies show that hypertensive individuals in low‐ and middle‐income countries, including Nigeria, do not actively observe self‐care to manage their conditions [7, 8, 9]. A study on 410 hypertensive adults registered for treatment at a healthcare center in Myanmar associated inadequate knowledge, poor diet, and non‐adherence to weight management and medications as major contributors to ineffective control of hypertension in a large population [10]. A community‐based cross‐sectional study in Kollam, India, reported low adoption of self‐care, which resulted from poor dietary, physical, and weight management approaches. This was observed to account for over 60% of the patient population not having optimal blood pressure control [11]. In Iran, a study by Eghbali et al. [12] revealed that high psychological distress negatively affects overall self‐care practice and blood pressure control among hypertensive patients. The study recommends psychological intervention in the self‐management of hypertension [12]. A mixed‐method survey on 402 hypertensive Pakistanis reported suboptimal blood pressure control attributed to inadequate knowledge of self‐care [13].

As in low‐ and middle‐income countries elsewhere, studies in some African countries also demonstrated the need for self‐care awareness and sensitization among patients with hypertension visiting healthcare facilities in some cities. While the East of Ethiopia reported an average level of self‐care [14], the West Ethiopian has a high prevalence of poor self‐care among hypertensive patients [15]. These studies linked occupation, comorbid conditions, socioeconomic status, and social support as major factors in self‐care practice outcomes. A qualitative study in Uganda on factors influencing health‐seeking behavior (HSB) among hypertensive patients recognized the need for strategies to address the multifactorial nature of HSB, as patients use various channels of care to manage hypertension. The study also pointed out that self‐medication without a prescription is common and it is a major contributor to poor self‐care practice [16]. Studies have shown that there is a high number of patients with uncontrolled blood pressure in Nigeria [17, 18, 19]. Previous studies examining the management of hypertension in Nigeria have less frequently focused on general self‐care practices [20]. Also, studies have identified that the prevalence of uncontrolled blood pressure is influenced by age, education, income, socioeconomic and sociodemographic characteristics, the nature of jobs [21], access to medical services, and level of adherence to medications [22]. Poor adherence to medication and general self‐care practices is positively correlated with poor blood pressure control rates [23].

There is a high prevalence of hypertension among adults in rural areas in Nigeria; these areas lack medical service centers, and there is no laid structure for monitoring and controlling the prevalence [24]. Following the emergence of COVID‐19, Nigeria has experienced a severe setback in healthcare delivery. A properly trained workforce in the sector leaves the country due to economic issues and underpayment, leading to a drastic decline in the deployment of medical staff to primary healthcare centers in semi‐urban and rural areas in Nigeria. Presently, consultation for hypertension management or treatment is mainly available at secondary and tertiary health centers, which are located primarily in urban settings across the country [25]. To our knowledge, limited reviews have delineated self‐care practices for hypertension across various African contexts, while also detailing the assessment techniques employed and the socio‐cultural aspects influencing behavior. This review employs a Preferred Reporting Items for Systematic Reviews and Meta‑Analyses extension for Scoping Reviews (PRISMA‑ScR)–aligned methodology to synthesize recent evidence regarding the prevalence and patterns of self‐care domains, including medication adherence, dietary practices (low salt), weight management, physical activity, alcohol consumption, smoking cessation, knowledge, and social support. It also emphasizes context‐specific barriers and facilitators pertinent to service design within African health systems. This review is relevant for summarizing the underlying factors that act as barriers and facilitators of general self‐care practice among patients with hypertension in Africa; hence, we conducted a scoping review of primary data from research carried out in Africa on self‐care of hypertension.

## 2 Methodology

This scoping review followed the PRISMA‑ScR framework, using guidance from the Joanna Briggs Institute (JBI) [26, 27]. The five‑stage methodological approach ensured transparency, reproducibility, and comprehensive coverage of the available evidence [28].

### 2.1 Stage 1: Literature Review Questions

Guided by the population‐concept‐context (PCC) framework, this review addressed two principal questions:

✓ What self‐care strategies do adults with hypertensive use to control their blood pressure in Africa?
✓ What factors are associated with self‐care practices among adults with hypertension in Africa?

### 2.2 Stage 2: Identifying

We applied a comprehensive, multilingual keyword strategy across six databases—CINAHL, PubMed/MEDLINE, ScienceDirect, PsycINFO, Embase, and Google Scholar. Search terms covered *self‐care*, *lifestyle modification*, *self‐management*, and *hypertension*, combined with African regional identifiers.

An initial search retrieved 8084 records, and an additional hand‐search helped ensure completeness. Keywords, databases accessed, and record counts are documented in Table 1 of the manuscript.

**Table 1:** Keywords for database and article identification.

Database	Search terms and keywords	Number of articles	Date accessed
CINAHL	Self‐care OR self‐management OR lifestyle management OR lifestyle management OR lifestyle modification AND hypertension OR high blood pressure OR blood pressure OR elevated blood pressure OR HTN OR hypertensive AND Africa OR sub‐Saharan Africa OR African metropolis OR African countries	109	November 6, 2024
PUBMED	Self‐care OR self‐management OR lifestyle management OR lifestyle management OR lifestyle modification AND hypertension OR high blood pressure OR Blood pressure OR elevated blood pressure OR HTN OR hypertensive AND Africa OR sub‐Saharan Africa OR African metropolis OR African countries	3988	November 6
ScienceDirect	Self‐care OR self‐management OR lifestyle management OR lifestyle modification AND hypertension OR hypertensive patients AND Africa	2878	November 6, 2024
PsycINFO	Self‐care OR self‐management OR self‐management OR lifestyle management OR lifestyle modification AND Hypertension OR Blood pressure OR high blood pressure OR elevated blood pressure OR HTN OR hypertensive AND Africa OR sub‐Saharan Africa OR African metropolis OR African countries	39	November 05, 2024
Embase	Self‐care OR lifestyle management OR lifestyle modification AND hypertension OR hypertensive patients	572	November 7
Google Scholar	Self‐care OR lifestyle management OR lifestyle modification AND hypertension OR Africa OR Nigeria	498	November 7

### 2.3 Eligibility Criteria

The inclusion criteria for this review were based on the PCC framework [27]. Quantitative, qualitative, and mixed‐method studies published from 2014 to 2024 were eligible; no mixed‐method studies met the criteria at full‐text (see Table 2). Study protocol articles, intervention studies, review articles, and eclampsia‐focused studies were exempted from this review. The review was limited to articles that were fully published in English.

**Table 2:** Inclusion criteria for the study using the PCC framework.

Criterion	Inclusion
Population	Hypertensive adults (18 years and over)
Concept	Qualitative, quantitative, and mixed‐method studies on the self‐care practice of hypertension
Context	African countries

### 2.4 Stage 3: Study Screening and Selection

**Figure 1:**
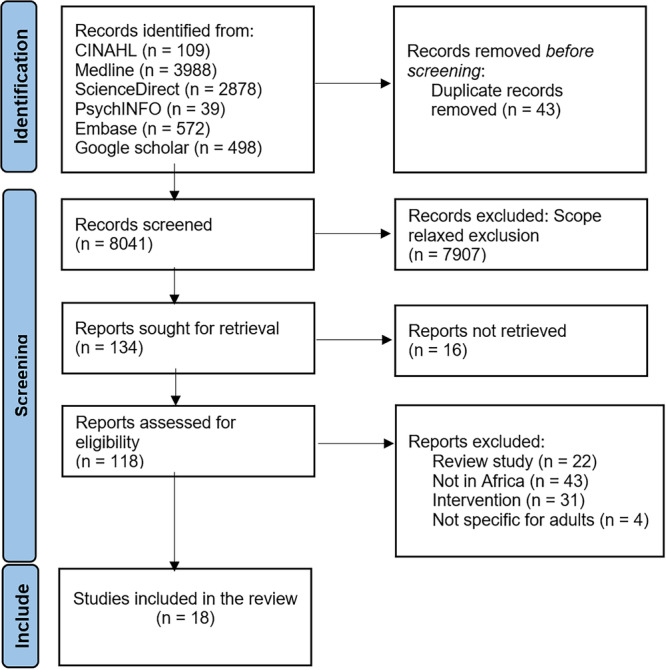
PRISMA flow diagram for scoping review on self‐care of hypertension in Africa. Flow chart adapted from [29].

Two reviewers independently screened titles, abstracts, and full texts against pre‐specified eligibility criteria. Disagreements were resolved by discussion or adjudication by a third reviewer. A PRISMA‐ScR flow diagram illustrates the study selection process, where 8041 records were screened, 134 reports were assessed for retrieval, 118 full texts were evaluated, and 18 studies met the inclusion criteria. Reasons for exclusion are documented within the PRISMA diagram (Figure 1).

### 2.5 Stage 4: Data Charting

We piloted a standardized extraction form and then charted data in duplicate, capturing study characteristics, setting, measurement instruments, and self‐care outcomes. Any uncertainties were resolved by consensus.

### 2.6 Stage 5: Collating, Summarizing, and Reporting Results

We used a descriptive synthesis. Studies were grouped by major self‐care domains. No statistical inference or meta‐analysis performed, in line with scoping review guidance. We used RefWorks for citation management and Microsoft Excel 365 for charting study characteristics and extracting domain‐specific self‐care outcomes. Themes were inductively developed from qualitative and quantitative findings [30], focusing on patterns, barriers, and facilitators of self‐care practices across African contexts.

### 2.7 Measurement of Self‐Care

Across the included studies, self‐care was measured using a combination of validated instruments, semi‐structured tools, and research developed questionnaire such as Hypertension self‐care activity level effects and its modification [13, 31, 32, 33], 8‐item Morisky Medication Adherence Scale (MMAS‐8) [22], and other informal tools that were context‐specific, tailored to local practice patterns [34, 35, 36].

### 2.8 Ethical Considerations

This scoping review synthesized previously published data. Therefore, ethical approval was not required.

### 2.9 Statistical Consideration

This study is a scoping review; therefore, no statistical analyses, hypothesis testing, subgroup analyses, or meta‐analyses were performed. No a priori levels of significance were defined, and no statistical software was used for analysis. Any statistical values (including *p*‐values, confidence intervals, or effect sizes) reported in Section Results, 3 reflect only the original authors' analyses and were not recalculated or reformatted for this review. Consistent with the Arksey and O'Malley framework, the JBI methodology, and PRISMA‐ScR guidance, data were synthesized descriptively to map the scope, characteristics, and nature of the existing evidence.

## 3 Results

### 3.1 Study Characteristics

Of the 18 articles included in this review, 8 were conducted in Ethiopia, 3 in Nigeria, 2 in Ghana, and 1 each in Tanzania, Uganda, Egypt, Malawi, and Tunisia. The majority of the studies were quantitative research, while three were qualitative research. None of the research uses a mixed‐method approach. The study included 4400 participants; 4289 enrolled in quantitative studies, with a minimum of 38 and a maximum of 422 participants per study. The total number of participants in the qualitative studies was 111. For more details on study characteristics, see Table 3.

**Table 3:** Articles included in the scoping review.

No	References	Country	Sample size	Settings	Study instruments
1	Adeola et al. [34]	Nigeria	298	Bi‐center teaching hospitals in the southwestern state	Quantitative study with a structured questionnaire
2	Ajiboye et al. [31]	Nigeria	38	Patients visiting the hospital outpatients in the city	Quantitative descriptive survey with H‐SCALE structured questionnaire
3	Gelaw et al. [32]	Ethiopia	392	Hospital‐based research	A cross‐sectional structured survey using the modified H‐Scale, ASAS‐R, MSPSS, and Knowledge of Hypertension Self‐care.
4	Gebremichael et al. [37]	Ethiopia	320,	Hospital‐based study	Quantitative study, cross‐sectional
5	Niriayo et al. [33]	Ethiopia	276	Ambulatory hypertensive patients visiting the Hospital	Quantitative cross‐sectional study
6	Bacha and Abera [38]	Ethiopia	384	Hospital‐based study	Quantitative cross‐sectional study
7	Ademe et al. [39]	Ethiopia	309	Public health institution	Quantitative cross‐sectional
8	Abdisa et al. [14]	Ethiopia	422	Multicentre study	Quantitative cross‐sectional study
9	Wake et al. [40]	Ethiopia	115	Hospital‐based	Quantitative cross‐sectional study
10	Tesfaye et al. [41]	Ethiopia	345	Hospital‐based study	Quantitative cross‐sectional
11	Tozivepi et al. [42]	Malawi	350	Hospital‐based	Quantitative cross‐sectional
12	Philbert et al. [43]	Tanzania	330	Hospital‐based	Quantitative, cross‐sectional study
13	Ketata et al. [44]	Tunisia	250	Multicentre primary health care	Quantitative cross‐sectional study
14	Elmasry [36]	Egypt	60	Hospital Outpatient	Quantitative
15	Anowie and Darkwa [35]	Ghana	400	Bicentre study	Quantitative cross‐sectional study
16	Nyaaba et al. [45]	Ghana	55	Community‐based	Qualitative cross‐sectional
17	Najjuma et al. [46]	Uganda	16	Hospital outpatient	Qualitative study
18	Odusola et al. [47]	Nigeria	40	Patients visiting primary healthcare	Qualitative study at a rural primary health care center

### 3.2 Self‐Care of Hypertension

Because the included studies used heterogeneous self‐care measurement approaches, ranging from validated tools (e.g., H‐SCALE, Hill‐Bone, MMAS‐8) to researcher‐developed questionnaires, the interpretation of pooled findings considered these methodological differences. In the quantitative studies included in this review, major studies reported medication adherence, knowledge about self‐management of hypertension, physical activity or exercise, alcohol intake, smoking cessation, and weight management [14, 32, 33, 34, 35, 37, 38, 39, 41, 42, 43, 44, 48, 49]. Social and family care, khat chewing, and co‐morbidity were reported in a few others. The aims and outcomes of the studies are presented in Table 4 below.

**Table 4:** Summary of the outcomes of included quantitative studies on self‐care of hypertension in Africa.

References	Design, *N*, tools	Overall self‐care outcomes	Sociocultural determinants and other outcomes
Abdisa et al. [14]	Cross‐sectional, 415, H‐SCALE	52% of hypertensive patients have adequate self‐care practices.	Medication adherence 50% Low salt intake 40% Physical activity 29.6% Smoking cessation/non‐smokers 85.8% Alcohol abstinence 50.2% Knowledge of hypertension 59.9%. Social support is 69%. Khat chewing 35%.
Ademe et al. [39]	Cross‐sectional, 309, Self‐care scale	More than half of the population of the participants (51%) have poor self‐care practices.	Divorced patients who lack a source of information have less chance of good self‐practice, while those with regular exercise, education, and spiritual counseling have good self‐care.
Adeola et al. [34]	Cross‐sectional, 298, Adapted developed structured questionnaire	Overall, self‐care practice was good in less than 14% of the patients.	There was low self‐integration and self‐management practices. 11.4% of the respondents adhered to medication, and 14.1% had a high practice of lifestyle modification.
Ajiboye et al. [31]	Cross‐sectional, 38, H‐Scale	Around 52.6% have poor lifestyle management practices.	There was poor knowledge and practice of lifestyle management among the participants.
Anowie and Darkwa [35]	Cross‐sectional, 400, Self‐developed questionnaire	Poor self‐efficacy commitment to treatment. Lack of education and knowledge.	There is poor knowledge of the cause, signs and symptoms, risk factors, prevention, and self‐care of hypertension among patients.
Bacha and Abera [38]	Cross‐sectional, 385, context‐specific informal tool	Good self‐care and adherence in 48.6% of patients. Good attitude towards control of hypertension in 39.5% of the participants.	Male sex, education, and being an urban resident are associated with knowledge, attitude, and good self‐care practice of hypertension among hospital patients.
Elmasry [36]	Cross‐sectional, 60, Hypertension knowledge level scale (HK‐LS), and Hypertension Self‐management Behavior Questionnaire (HSMBQ)	55% have unsatisfactory knowledge of self‐management. There is rampant negative behavior and negligence regarding hypertension self‐care and management.	Knowledge and self‐management behaviors among the subjects were unsatisfactory.
Gebremichael et al. [37]	Cross‐sectional, 320, H‐SCALE	Overweight, comorbidity, non‐adherence to anti‐hypertensive medication, non‐adherence to physical activity, and non‐adherence to alcohol abstinence are predictors of uncontrolled hypertension.	Uncontrolled hypertension in 52.5%, with a high prevalence of non‐adherence to physical activity, alcohol abstinence, and medication.
Gelaw et al. [32]	Cross‐sectional, 392, H‐SCALE	The self‐care practice among hypertension patients was found to be 54.1%.	Urban residency, social support, good knowledge, and age above 40 were positively correlated with good self‐care practice. Overall, good self‐care practice in 54.1% of the participants.
Ketata et al. [44]	Cross‐sectional, 250, multidimensional self‐administered questionnaire for hypertension self‐care	More than 50% of the participants have a good self‐care score.	Adults above 65, University education, and health education were noted to be positively influenced by good self‐care practice.
Niriayo et al. [33]	Cross‐sectional, 276, H‐SCALE	48.2% were adherent to medication, while 21.5% and 29% adhered to weight management and low salt intake, respectively.	There was low adherence to self‐care behavior, particularly weight management, low salt intake, exercise, and medication among participants.
Philbert et al. [43]	Cross‐sectional, 330, Hypertension Evaluation of Lifestyle Modification (HELM)	Good self‐care of hypertension was reported in 19.7%.	Education level, having a family member with a history of hypertension, and knowledge of self‐care practice were markedly linked with good self‐care practice.
Tesfaye et al. [41]	Cross‐sectional, 345, H‐SCALE	Lack of awareness of hypertension‐related complications, non‐adherence to smoking abstinence, non‐adherence to alcohol abstinence, Khat (Catha edulis) chewing, overweight, middle age, and old age were significant predictors of uncontrolled hypertension.	A high prevalence of uncontrolled hypertension was associated with an unhealthy lifestyle. Continuous awareness of lifestyle practices in hypertension‐related complications during follow‐up visits by healthcare providers is recommended.
Tozivepi et al. [42]	Cross‐sectional, 350, Adopted WHO survey	About 5.1% of the respondents were reported to adhere to all the recommended lifestyle behaviors.	There were low adherence rates reported for medication, diet, and physical activity. The study further revealed that participants who adhered to antihypertensive treatment and alcohol cessation had reduced odds of having uncontrolled hypertension, while consuming deep‐fat fried foods caused threefold odds of uncontrolled BP. Reducing dietary salt reduces the odds of uncontrolled hypertension.
Wake et al. [40]	Cross‐sectional, 115, context‐specific informal tool	There was a high level (67.0%) of non‐adherence to self‐care practices.	Despite higher medication adherence, age, formal education, and length of diagnosis are significantly linked with the level of self‐care practice.

Qualitative studies that were included explored inhibitors and facilitators for adhering to antihypertensive pharmacotherapy [47], medication adherence among patients [46], and coping strategies developed and adopted in managing hypertension [45]. Findings from the quantitative studies are presented in Table 5 below.

**Table 5:** Summary of the outcomes of included qualitative studies of self‐care of hypertension in Africa.

References	Design, *N*, tools	Research focus	Outcome summary
Odusola et al. [47]	Qualitative interview, 40, inductive thematic coding and analyses	Income and health insurance on adherence to hypertension treatment	Negative perceptions of decreased body weight and size, incompetent clinical operations, high salt intake, non‐adherence to the medication regimen, and use of herbal supplements were notable hindrances associated with self‐care practice
Najjuma et al. [46]	One‐on‐one in‐depth interviews, 16, narrative data with thematic analyses	Adherence and non‐adherence to the management of hypertension	Lack of medical supply, use of self‐prescription, and stigma were reported to influence self‐care efficacy and practice
Nyaaba et al. [45]	Cross‐sectional qualitative, 55, semi‐structured interview	How social and physical context influence patience perception of hypertension and their coping strategies	Rural non‐immigrants use traditional medicine or remedies to mitigate expensive medication and search for alternative treatments for hypertension. An indication of poor self‐care practice

## 4 Discussion

This scoping review highlights the persistent challenges surrounding effective self‐care practices among adults in Africa. Despite robust evidence supporting the benefits of effective self‐care behaviors for blood pressure control, this review demonstrated that there is poor self‐care practice among patients with hypertension in Africa. The self‐care inadequacy in medication adherence, lifestyle modification, and routine monitoring is blamed on medical outreach, lack of trust in the efficacy of orthodox medication compared to traditional or herbal concoctions, insufficient health providers, and negligence in weight reduction, which were identified as barriers against effective self‐care practice from the study. The key finding from this synthesis is the interplay between knowledge, sociodemographic characteristics, and sociocultural influences, which collectively shape individuals' self‐care practices [34, 36, 38, 44].

The need for education and awareness towards self‐care management of hypertension cannot be undermined. For instance, one Nigerian study showed poor knowledge and low practice of lifestyle management among hypertensive adults [34], while an Ethiopian study found that only about half of participants possessed adequate knowledge to support positive self‐care behaviors [14]. Bacha and Abera linked education and demographic characteristics of patients with good self‐care practice, attributing urban settlement to good self‐care practice [38]. In addition, patients with family members with initial experience were reported to influence their way of managing hypertension through educating their family, giving personal relationship experiences, and social support and awareness on diagnosis and management of hypertension, which was reported to influence their self‐practice positively [43]. Poor knowledge about hypertension and polypharmacy‐related management was documented to affect self‐care practice negatively [50]. These findings highlight the need for effective education and awareness in the cause of managing hypertension.

Sociocultural factors also emerged as central determinants of self‐care behavior. Across included studies, cultural norms around body weight, faith‐based medication adjustments, perceptions of traditional remedies, and stigma related to illness influenced how individuals interpreted hypertension and adhered to treatment. For instance, practices such as khat chewing, reliance on herbal preparations, or spiritual interpretations of illness were noted to undermine adherence to biomedical recommendations. Studies highlighted cultural norms affecting attitudes toward weight loss, herbal remedies, and medication. For example, qualitative evidence revealed patients' reluctance to lose weight due to stigma, faith‐based modifications of medication regimens [31], and reliance on traditional herbal concoctions over prescribed antihypertensives [31, 35]. In Uganda, limited drug supply and the resulting reliance on self‐prescription further underscored how cultural and health system constraints intertwine to influence adherence [43]. The practice of khat chewing in Ethiopian communities was also noted as a culturally embedded behavior hindering effective self‐care [14, 40]. Similarly, stigma associated with weight loss or chronic illness was reported to discourage sustained lifestyle changes [32, 39]. These findings underscore that self‐care practices cannot be fully understood without considering the social and cultural environments in which individuals live. Strong social support was strongly linked with good self‐care practice and adequate blood pressure control [14, 32, 34, 39]. Social support is another predictor of self‐care; surprisingly, few of the included studies explored the influence of social support on the self‐care practice of patients. As an integral part of the self‐care plan, the WHO recommends the need for effective social support in coping with chronic conditions like hypertension [51].

Non‐adherence to medication is accompanied by uncontrolled hypertension, serious complications, and limitations of healthcare resources. Medication adherence was a mixed outcome from this study. while one of the studies recorded a high rate of medication adherence in a population with two‐thirds having formal education [40]. There was poor adherence to medication in other studies, which is attributed to several factors, such as a lack of trust in the efficacy of Orthodox, and the cost of medication, which invariably shifts the attention of the patients to herbs and traditional medicine [31, 33, 35, 41, 42, 47]. A shift in attention towards the use of traditional medicine as an alternative was also reported in Asia [52]. Structural and systemic barriers were particularly prominent. Medicine shortages, limited access to healthcare, and inadequate counseling were reported as major obstacles to proper self‐care. Ethiopian and Ugandan studies repeatedly noted drug stock‐outs and inconsistent provider support as key factors leading to non‐adherence and poor control [37, 38, 39, 43]. Inadequate health workforce availability and long distances to clinics further limited patients' engagement with recommended practices, as indicated in qualitative work from Uganda and multiple Ethiopian hospital studies [32, 37, 46].

Lifestyle‐related self‐care domains—particularly physical activity, dietary modification, and weight management—showed consistently low adherence across countries [3, 19, 23, 33]. Although exercise is a well‐established strategy for blood pressure reduction [53], participants from the selected studies often neglect exercise due to misconceptions, lack of facilities, or awareness [31, 33, 35, 41, 42, 47]. In a study by NHANES, where participants were counseled for physical activity, 71% of those who followed the recommendation by the physician had a significant decline in blood pressure [54]. Facilitating physical activity requires raising awareness and planning the environment, such as creating a convenient community place for exercise and fostering effective self‐monitoring and self‐efficacy in achieving daily aerobic activities.

Conclusively, the level of self‐care practice among hypertensive patients remains poor among Africans visiting hospital facilities in major cities. Studies revealed that sociodemographic determinants that majorly influence the level of self‐care practice among patients with hypertension are formal education, level of income, and urban or rural residence. These characteristics play pivotal roles in awareness and inclination to medical visits for diagnoses and management of hypertension. Invariably, the disparities in the sociodemographic characteristics affect the level at which patients effectively and collectively adhere to the domains of self‐care practice. One of the included studies reported that urban settlement had a positive influence on the level of self‐care practice; however, the majority of studies on self‐care for hypertension in Africa targeted patients visiting major referral hospitals in urban areas. This may not give a detailed representation of the level of self‐care practice of patients with hypertension in the rural communities.

## 5 Limitation

The original strategy encompassed an urban–rural comparison; however, this was unfeasible as the bulk of the included studies lacked stratified data by setting. Future primary studies should report contextual characteristics due to their significance for health service planning in Africa.

## Author Contributions

**Abiola Adeosun:** conceptualization, investigation, methodology, software, data curation, writing – original draft, formal analysis, project administration, resources, funding acquisition. **Hafiz T. A. Khan:** writing – review and editing, supervision, visualization, formal analysis, project administration, resources, funding acquisition. **Muili Lawal:** supervision, writing – review and editing, visualization, resources, validation, funding acquisition. All authors contributed equally, and all read and approved the final article.

## Conflicts of Interest

The authors declare no conflicts of interest.

## Transparency Statement

The lead author, Abiola Adeosun, affirms that this manuscript is an honest, accurate, and transparent account of the study being reported; that no important aspects of the study have been omitted; and that any discrepancies from the study as planned (and, if relevant, registered) have been explained.

## Supporting information

Supporting File

## Data Availability

Data sharing is not applicable to this article as no datasets were generated or analyzed during the current study. This scoping review does not contain any primary data; no new data were created. The data supporting the findings of this study are derived from published sources, which are cited within the article.
